# Characterization of the complete chloroplast genome of the Tangut monkshood *Aconitum tanguticum* (Ranunculales: Ranunculaceae)

**DOI:** 10.1080/23802359.2020.1773338

**Published:** 2020-06-02

**Authors:** Qien Li, Xianjia Li, Rangzhong Qieyang, Cairang Nima, Duojie Dongzhi, Duojie, Xiao Guo

**Affiliations:** a Tibetan Medicine Research Center, Tibetan Medical College, Qinghai University, Xining, People’s Republic of China; b State Key Laboratory of Tibetan Medicine Research and Development, Qinghai University, Xining, People’s Republic of China

**Keywords:** Iterative mapping, phylogenetic analysis, plastid genome, Tangut monkshood, *Aconitum tanguticum*

## Abstract

The Tangut monkshood (*Aconitum tanguticum*) is a perennial herb with high medicinal values. Here, its chloroplast genome was assembled from Illumina sequencing reads. The circular genome is 157,114 bp long with an A + T-biased nucleotide composition, and comprises a pair of inverted repeat (IR) regions (26,255 bp), separated by a large single-copy (LSC) region (87,559 bp) and a small single-copy (SSC) region (17,045 bp). It encodes a total of 112 gene species, with 19 of them being completely or partially duplicated and 18 of them harboring one or two introns. Phylogenetic analysis recovered two major clades of the genus *Aconitum*.

*Aconitum tanguticum*, commonly known as Tangut monkshood, is a perennial herb within the family Ranunculaceae (Ranunculales), and is mainly distributed in Gansu, Qinghai, Shaanxi, Sichuan, Tibet and Yunnan Provinces of China with an elevation of 3200–4800 m (Li and Yuichi 2001). This herb has long been used in traditional Tibetan medicine for treating gastritis, hepatitis, influenza, nephritis, pneumonia and other diseases (Nanjing University of Traditional Chinese Medicine 2005). To date, most studies of *A. tanguticum* have been focused upon its phytochemistry (e.g. Xu et al. 2013; Li et al. 2014, 2015). Little is known about its genomics. In this study, its complete chloroplast genome was assembled from high-throughput Illumina sequencing reads. The annotated sequence was deposited into GenBank under the accession number MT430949.

Fresh leaves of a single individual were collected from Laji Mountain (101°47′11″E, 36°02′14″N) with the voucher specimen deposited in Qinghai University (accession number: LQE-2019-066), and were used for genomic DNA extraction with the DNeasy Plant Mini Kit (Qiagen, CA, USA). The high-throughput DNA sequencing was performed on the Illumina HiSeq X Ten Sequencing System (Illumina, CA, USA), and yielded a total of 94.97 M of 150-bp raw paired reads. The chloroplast genome was assembled using MITObim v1.9 (Hahn et al. 2013) with that of *Aconitum carmichaelii* (Yang et al. 2018) as the initial reference. Genomic annotation was done in Geneious Prime 2020 (Biomatters Ltd., Auckland, New Zealand) by aligning with those of its congeners, e.g. *Aconitum delavayi* (MG678802) (Meng et al. 2018), *Aconitum chiisanense* (KT820665) (Lim et al. 2017) and *Aconitum reclinatum* (MF186593) (Kong et al. 2018).

The chloroplast genome of *A. tanguticum* is 157,114 bp in size, and comprises a pair of inverted repeat (IR) regions (26,255 bp), separated by a large single-copy (LSC) region (87,559 bp) and a small single-copy (SSC) region (17,045 bp). The nucleotide composition is asymmetric with an overall A + T content of 62.0% (‘light strand’). In all, 112 gene species were annotated, including 78 protein-coding (PCG), 30 tRNA and four rRNA gene species. Nineteen gene species are completely or partially duplicated, including eight PCGs (*ndhB*, *rpl2*, *rpl23*, *rps7*, *rps12*, *ycf1*, *ycf2* & *ycf15*), seven tRNAs (*trnA-UGC*, *trnI-CAU*, *trnI-GAU*, *trnL-CAA*, *trnN-GUU*, *trnR-ACG* & *trnV-GAC*) and all four rRNAs (*rrn4.5*, *rrn5*, *rrn16* & *rrn23*). Besides, one or two introns are present in 18 gene species (i.e., *atpF*, *clpP*, *ndhA*, *ndhB*, *petB*, *petD*, *rpl2*, *rpl16*, *rpoC1*, *rps12*, *rps16*, *ycf3*, *trnA-UGC*, *trnG-GCC*, *trnI-GAU*, *trnK-UUU*, *trnL-UAA* and *trnV-UAC*).

A Bayesian phylogeny was reconstructed using chloroplast PCGs for a panel of 24 species within the genus *Aconitum* with the program MrBayes v3.1.1 (Huelsenbeck and Ronquist 2001; Ronquist and Huelsenbeck 2003) (Figure 1). ‘GTR + G+I’ was selected as the best-fit nucleotide substitution model by TOPALi v2.5 (Milne et al. 2009). Three species within the genus *Delphinium*, i.e. *D. maackianum* (MN648402) (He et al. 2019), *D. anthriscifolium* (MK253461) and *D. ceratophorum* (MK253460) (Park et al. 2020), were included as outgroup taxa. All 24 species were clustered into two major clades, with one clade consisting of seven species (*A. angustius*, *A. barbatum*, *A. finetianum*, *A. longecassidatum*, *A. pseudolaeve*, *A. reclinatum* & *A. sinomontanum*) and the other consisting of the remaining 17 species. *A. tanguticum* was placed within the latter larger clade.

**Figure 1.**
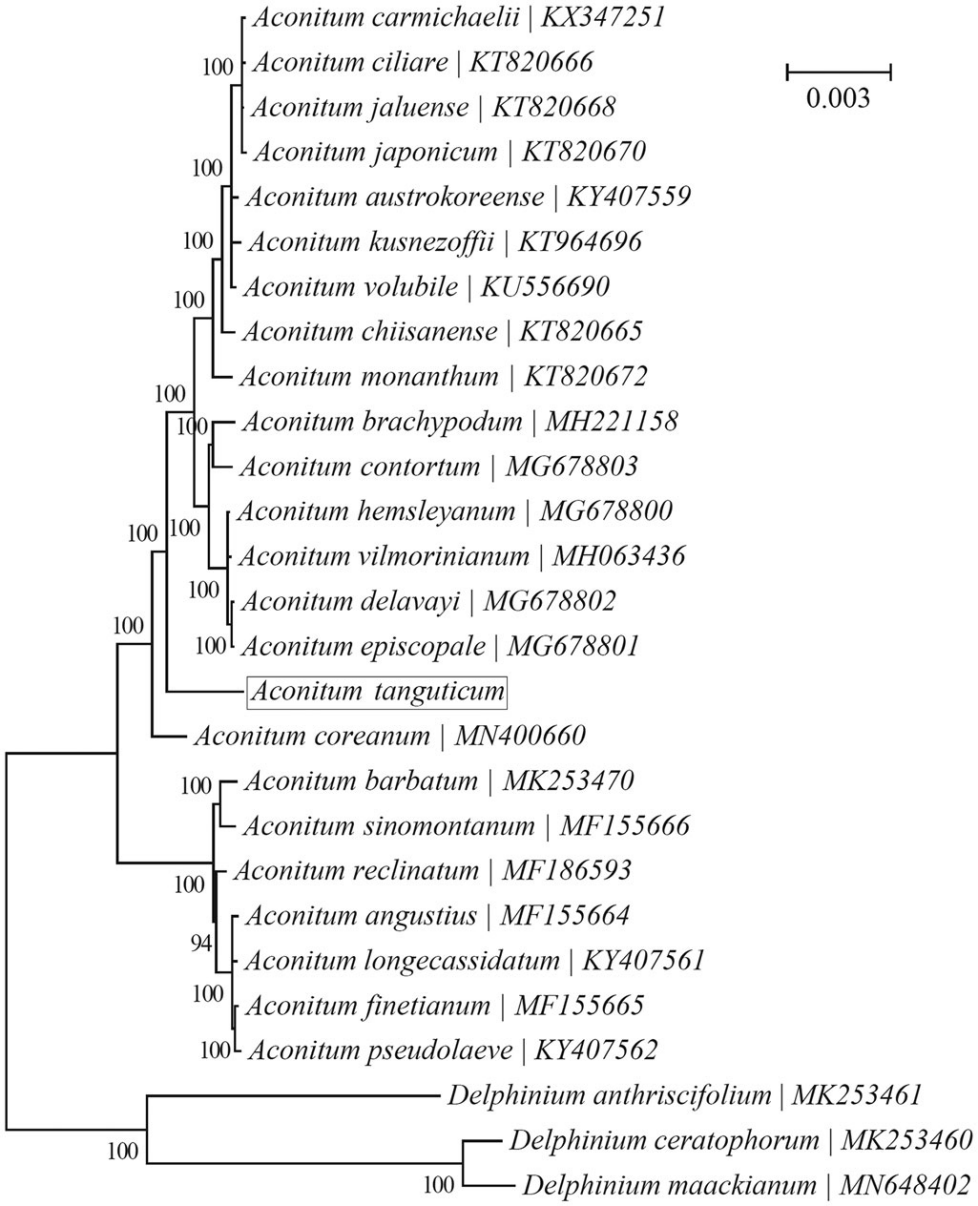
Phylogenetic relationships of 24 species within the genus *Aconitum* based on the Bayesian analysis of the concatenated coding sequences of chloroplast PCGs. The best-fit nucleotide substitution model is ‘GTR + G+I’. Three contribal species within the genus *Delphinium* were included as outgroup taxa.

## Data Availability

The data that support the findings of this study are openly available in GenBank of NCBI at https://www.ncbi.nlm.nih.gov, reference number MT430949.
